# Demographic and clinical profile of an inception cohort of road trauma survivors

**DOI:** 10.1186/s12889-023-16487-w

**Published:** 2023-08-12

**Authors:** Lulu X. Pei, Herbert Chan, Leona K. Shum, Lina Jae, John A. Staples, John A. Taylor, Devin R. Harris, Jeffrey R. Brubacher

**Affiliations:** 1 Department of Emergency Medicine, University of British Columbia, Vancouver, Canada; 2 Department of Medicine, University of British Columbia, Vancouver, Canada

**Keywords:** Cyclist, Pedestrian, Motorcyclist, Motorist, Road trauma, Injury severity, Health-related quality of life, Emergency department

## Abstract

**Background:**

Road trauma is a major public health concern, often resulting in reduced health-related quality of life and prolonged absenteeism from work even after so-called ‘minor’ injuries that do not result in hospitalization. This manuscript compares pre-injury health, sociodemographic characteristics and injury details between age, sex, and road user categories in a cohort of 1,480 road trauma survivors.

**Methods:**

This was a prospective observational inception cohort study of road trauma survivors recruited between July 2018 and March 2020 from three trauma centres in British Columbia, Canada. Participants were aged ≥ 16 years and arrived in a participating emergency department within 24 h of involvement in a motor vehicle collision. Data were collected from structured interviews and review of medical records.

**Results:**

The cohort of 1,480 road trauma survivors included 280 pedestrians, 174 cyclists, 118 motorcyclists, 683 motor vehicle drivers, and 225 passengers. Median age was 40 (IQR = [27, 57]) years; 680 (46%) were female. Males and younger patients were significantly more likely to report better pre-injury physical health. Motorcyclists and cyclists tended to report better physical health and less severe somatic symptoms, whereas pedestrians and motor vehicle drivers reported better mental health. Injury severity and hospital admission rates were higher in pedestrians and motorcyclists and lower in motorists. Upper and lower extremity injuries were most common in pedestrians, cyclists and motorcyclists, whereas neck injuries were most common in motor vehicle drivers and passengers.

**Conclusions:**

In a large cohort of road trauma survivors, overall injury severity was low. Motorcyclists and pedestrians, but not cyclists, had more severe injuries than motorists. Extremity injuries were more common in vulnerable road users. Future research will investigate one-year recovery outcomes and identify risk factors for poor recovery.

**Supplementary Information:**

The online version contains supplementary material available at 10.1186/s12889-023-16487-w.

## Background

Each year in Canada, road trauma causes approximately 150,000 injuries including over 1,900 fatalities and 9,400 serious injuries [1]. The annual cost of road trauma in Canada is uncertain, with estimates ranging from $4.3 billion [2] to $62.9 billion [3]. In addition to the economic cost, injury-related disability is a major public health concern [4, 5]. Chronic pain and psychological problems such as post-traumatic stress disorder (PTSD) are more common following road trauma than after other injuries [6] perhaps due to poor expectations of recovery following the road trauma. Most road trauma research focuses on severe injuries which can be life-threatening or result in permanent disability. However, minor injuries, which constitute the majority of road trauma cases, are also associated with substantial health care costs and can result in reduced health-related quality of life (HRQoL) and prolonged absenteeism from work [7–9].

Previous road trauma outcome research often focuses on motorists (motor vehicle drivers and passengers) and excludes cyclists and pedestrians [10–25]. However, active transport in the form of walking and cycling is gaining popularity as a healthy means of commuting [26]. In addition to the environmental benefits of reducing greenhouse gas emissions and air pollution, active transport has numerous health benefits including reduced risk of obesity, cardiovascular disease, diabetes, hypertension and cancer [27]. Unlike motorists, cyclists and pedestrians are not protected by the safety features of a vehicle and are considered vulnerable road users. They are at risk of severe injury in the event of a motor vehicle collision and may have different injury profiles and recovery trajectories following road trauma.

In addition, previous cohort studies enrolled road trauma survivors weeks after the incident at the time they filed insurance claims, [13–19, 28–30] which can lead to selection bias by excluding those who recover quickly and do not file insurance claims. Enrolment delays may also lead to recall bias. Other studies excluded patients with minor injuries that did not require hospital admission [31] and people who did not speak the dominant language [7, 13–15, 17, 18, 28, 32–41]. Many risk factors for poor recovery are likely related to cultural factors; at present, most road trauma cohort studies have been conducted in Europe or Australia with relatively limited data from North America. To address limitations of previous research, we conducted an inception cohort study of almost 1,500 road trauma survivors who were injured in a motor vehicle collision in British Columbia, Canada. We included all patients who presented to the emergency department for road trauma injuries, including those with less serious injuries who were discharged home directly from the emergency department. We did not capture patients who did not require emergency department treatment. This manuscript reports pre-injury health, sociodemographic factors and the type, location and severity of injuries for this cohort. Our aim is to compare sociodemographic factors, pre-injury health, and injuries between males vs. females, age groups, and road user types.

## Methods

### Study setting and recruitment

This was a prospective observational study of an inception cohort of road trauma survivors, recruited between July 2018 and March 2020 from three British Columbia trauma centres: Vancouver General Hospital (Vancouver), Royal Columbian Hospital (New Westminster), and Kelowna General Hospital (Kelowna). We recruited cyclists, pedestrians, motorcyclists, motor vehicle drivers and motor vehicle passengers. This study was approved by the research ethics board of the University of British Columbia. Participants provided informed written or verbal consent. For minors (16–18 years old), parental/guardian permission was obtained in addition to participant assent. For participants unable to provide consent (e.g., comatose), proxy consent was obtained from a designated caregiver. Detailed methods for recruitment and study procedures have been published previously [42].

### Eligibility

Road trauma survivors aged 16 years and older who arrived in a participating emergency department (ED) within 24 h of a collision involving at least one motorized vehicle were included. Injuries not involving a moving motorized vehicle were excluded. Children younger than 16 years old were excluded due to differing recovery trajectories and tools required to measure health-related quality of life. Cognitively impaired survivors were included if consent and study information could be obtained from a reliable proxy such as a partner or parent. Non-English speakers were interviewed by a multilingual research assistant or through a translator when available. Fatalities within 30 days following the hospital visit or admission were excluded.

### Outcome measures

Data were collected from baseline interviews and review of medical records. In addition to English, interviews were also conducted in Cantonese, French, Korean, Mandarin, Punjabi, and Vietnamese to include non-English speaking participants (reflecting the common languages spoken in greater Vancouver). Baseline interviews were conducted as soon as possible following crash, within 7 days in most cases, and allowed for determination of pre-existing health and functional status as well as other potential risk factors for a poor recovery outcome. Data collected included demographic and socioeconomic information (e.g., age, sex, education level, ethnicity), crash details (such as road user type), as well as injury details such as injury location, injury severity score and degree of pain. We used validated instruments to assess pre-event anxiety and depression with the Patient Health Questionnaire-4 (PHQ-4; two weeks prior to accident), [43, 44] somatic symptoms experienced in the four weeks prior to injury with the PHQ-15, [45] and pain catastrophizing and coping with the Pain Catastrophizing Scale (PCS; before the accident) [46]. Pre-injury health-related quality of life was assessed with the five-level EuroQol instrument and visual analogue scale (day before injury) as well as the 12-Item Short Form Health Survey (SF-12; four weeks prior to event). These validated tools assess mental health (depression, anxiety), discomfort and pain, and limitations to daily life activities (bending or lifting, ambulation, self-care, and daily and social activities).

Total scores for each instrument were calculated according to the instrument user manuals or scoring software. For partially completed responses, missing values were handled following the guidelines set out by each validated tool to obtain a total outcome score. For instruments without documentation on how to handle missing responses, total scores were standardized based on the proportion of total response items within those instruments that were reported by the participant. Cut-offs for mild, moderate, and severe symptom categories for each instrument were referenced from the respective user manuals: a PCS score of 30 or more represents clinically relevant catastrophizing; [46] PHQ-15 scores of 5, 10, and 15 represent cutpoints for low, medium, and high somatic symptom severity, respectively; [47] PHQ-4 scores of 0–2, 3–5, 6–8, and 9–12 indicate no, mild, moderate, and severe psychological distress [44]. SF-12 summary scores were calculated using the proprietary PRO CoRE software which includes a missing data estimation algorithm for handling incomplete responses [48]. SF-12 physical and mental component scores are standardized based on 1998 U.S. population norms to have a mean of 50 and standard deviation of 10 [48]. Lower scores suggest poorer physical or mental health (e.g., limitations in physical functioning for the physical component; frequent psychological distress for the mental component).

The medical records of the index hospital visit for all participants were reviewed and served as the sole source of information for injury severity and type and need for hospital admission. When missing from the baseline interview, the medical record provided information on accident details, road user type, injury type and location, medical history or medication use; the baseline interview was considered the reference standard for this information in case of discrepancies between baseline interview and medical chart review.

Outcome measures were summarized descriptively using mean and standard deviation for continuous variables and count and proportion for categorical variables. Outcome measures were disaggregated by sex, age groups, and road user types and compared across groups using chi-square test (or Fisher’s exact test) for categorical variables and t-test or ANOVA test for continuous variables.

## Results

**Fig. 1:**
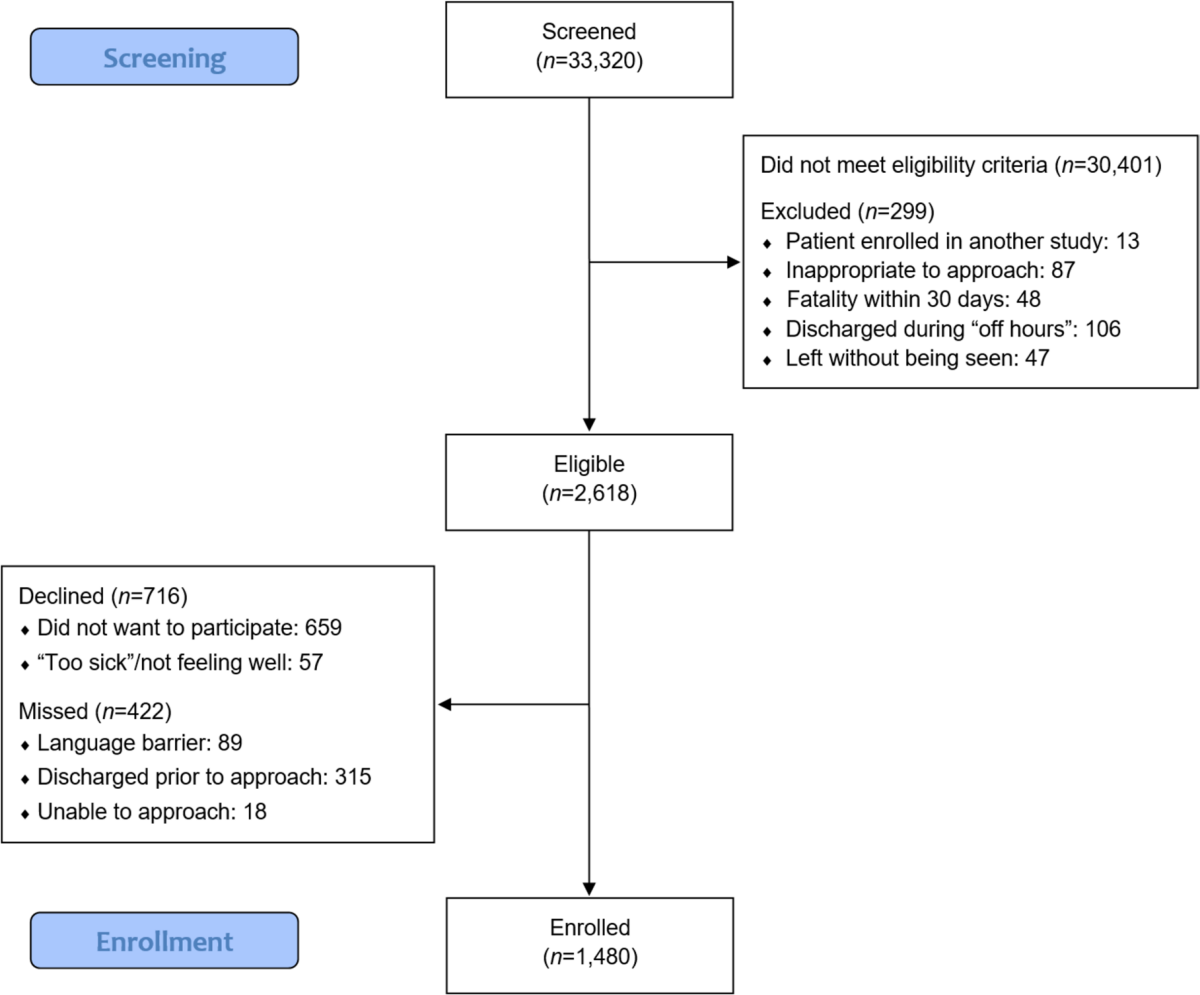
Flow diagram of screened and enrolled participants included in the present analysis

During the study period, 2,618 road trauma survivors were eligible and 1,480 (56.5%) were enrolled (Fig. 1). Refusal rates were higher in the smaller hospital sites (RCH and KGH). Eligible individuals who refused participation were older (median [IQR] age = 44 [30, 60] years) than those who participated (median [IQR] age = 40 [27, 57]; *p* < 0.001) but there was no difference with respect to the proportion of males and females. There was also a significant difference in distribution of road user types; a greater proportion of refusals were drivers compared to enrolled participants and a smaller proportion were cyclists. No differences were observed regarding ED disposition between refusals and enrolled participants (Supplementary Table S1). Baseline interviews for eligible individuals consenting to participate were conducted within 7 days following crash in 92.8% of cases (*n* = 1,374/1,480).

Supplementary Table S2 summarizes sociodemographic characteristics of the cohort. Of the 1,480 enrolled participants, 680 (46%) were female and the median age was 40 (IQR = 27–57) years with almost half (43.6%) aged ≤ 35 years. Over half of the cohort had a post-secondary degree; post-secondary education was more common among females. Most participants were employed (66.3%), with a higher proportion of employment in males (72.8%) than females (58.7%; *p* < 0.001). Half of the participants were Caucasian. Among the enrolled participants, 10.3% (*n* = 153/1,480) spoke limited English and required a translator.

**Fig. 2:**
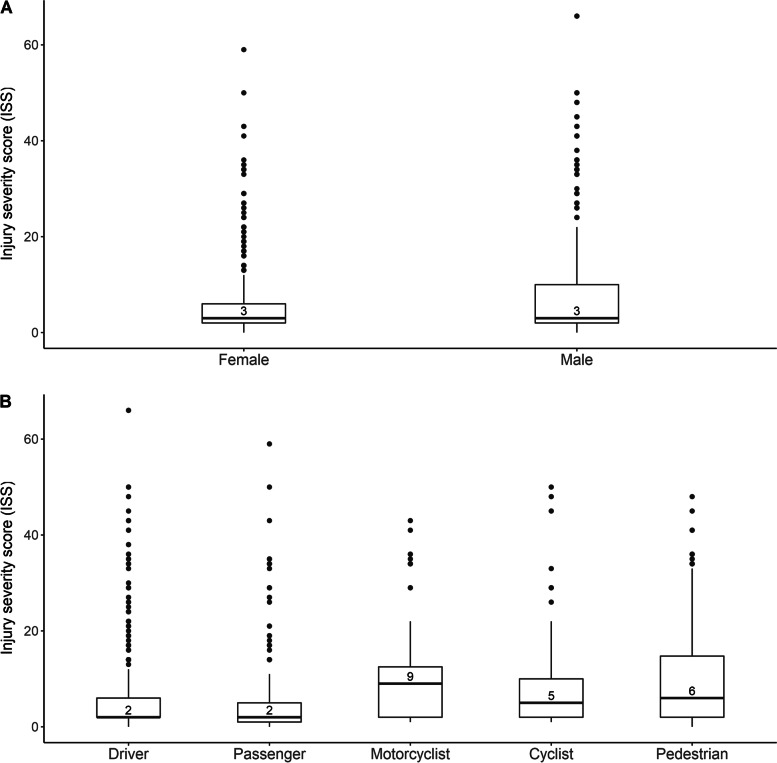
Side-by-side boxplots of injury severity score (ISS) by sex and road user type

Table 1 summarizes ED visit details, distribution of road user types, injury symptoms and recovery expectations for males and females. Across the three participating emergency departments, the majority of participants were recruited from VGH (> 80%), the largest hospital site. One quarter of all recruited participants (24.1%) were admitted to hospital. As expected, most participants were motor vehicle drivers (46.1%) or passengers (15.2%). Compared to males, a higher proportion of female participants were passengers (9.5% *vs.* 21.9%; *p* < 0.001) and a lower proportion were motorcyclists (12.4% *vs.* 2.8%; *p* < 0.001) or cyclists (16.6% *vs.* 6.0%; *p* < 0.001). The most commonly experienced symptoms were neck and back pain/stiffness, with ~ 40% of participants reporting these symptoms. Symptoms of headache and shortness of breath experienced immediately following the accident were more commonly reported among females compared to males. Relative to males, females reported higher pain severity scores (mean pain severity 6.71 *vs.* 6.18 for males; *p* < 0.001) but had lower injury severity scores (mean ISS = 5.75 *vs.* 8.26 for males; *p* < 0.001) and were less likely to be admitted to hospital (18.2% *vs*. 29.0% for males; *p* < 0.001). Median ISS was the same for males and females; however, some males had more extreme values thereby inflating the mean ISS for males (Fig. 2). In terms of recovery expectations, most participants were unsure of their recovery trajectory (43%, *n* = 498), with most others expecting recovery to take less than 1 week (11.8%, *n* = 137), between 1 week and 1 month (22.3%, *n* = 258), or between 1 and 3 months (10.9%, *n* = 126). No significant differences in recovery expectations were observed between males and females.

**Table 1:** Emergency department visit details, post-injury pain, symptoms, and recovery expectations^1^

	All (*n* = 1,480)	Sex		
		Female (*n* = 680)	Male (*n* = 800)	*P*-value^2^
Site				
VGH	1,219 (82.4%)	553 (81.3%)	666 (83.3%)	*p* = 0.107
RCH	219 (14.8%)	101 (14.9%)	118 (14.8%)	
KGH	42 (2.8%)	26 (3.8%)	16 (2.0%)	
ED discharge disposition				
Discharged home	1,105 (74.7%)	547 (80.4%)	558 (69.8%)	*p* < 0.001
Admitted to hospital	356 (24.1%)	124 (18.2%)	232 (29.0%)	
Left against medical advice / without being seen / before treatment completed	15 (1.0%)	6 (0.9%)	9 (1.1%)	
Road user type				
Driver	683 (46.1%)	332 (48.8%)	351 (43.9%)	*p* < 0.001
Passenger	225 (15.2%)	149 (21.9%)	76 (9.5%)	
Motorcyclist	118 (8.0%)	19 (2.8%)	99 (12.4%)	
Pedestrian	280 (18.9%)	139 (20.4%)	141 (17.6%)	
Cyclist	174 (11.8%)	41 (6.0%)	133 (16.6%)	
Injury severity score (ISS)	7.11 (9.37)	5.75 (7.67)	8.26 (10.50)	*p* < 0.001
Symptoms immediately after accident^3^				
Headache	425 (28.7%)	225 (33.1%)	200 (25.0%)	*p* < 0.001
Chest pain	313 (21.1%)	159 (23.4%)	154 (19.3%)	*p* = 0.061
Back pain/stiff back	566 (38.2%)	261 (38.4%)	305 (38.1%)	*p* = 0.962
Neck pain/stiff neck	585 (39.5%)	282 (41.5%)	303 (37.9%)	*p* = 0.175
Shortness of breath	383 (25.9%)	198 (29.1%)	185 (23.1%)	*p* = 0.010
Dizziness	489 (33.0%)	237 (34.9%)	252 (31.5%)	*p* = 0.190
Other	1,218 (82.3%)	557 (81.9%)	661 (82.7%)	*p* = 0.772
Pain severity (0–10)^4^	6.43 (2.48)	6.71 (2.57)	6.18 (2.38)	*p* < 0.001
Recovery expectation^4^				
< 1 week	137 (11.8%)	63 (11.7%)	74 (12.0%)	*p* = 0.363
1 week to < 1 month	258 (22.3%)	122 (22.6%)	136 (22.0%)	
1 month to < 3 months	126 (10.9%)	58 (10.7%)	68 (11.0%)	
3 months to < 6 months	59 (5.1%)	20 (3.7%)	39 (6.3%)	
≥ 6 months	78 (6.7%)	33 (6.1%)	45 (7.3%)	
Don’t know	498 (43.0%)	243 (45.0%)	255 (41.3%)	

^1^Values are *n* (column %) or mean (SD). VGH, Vancouver General Hospital; RCH, Royal Columbian Hospital; KGH, Kelowna General Hospital

^2^P-value obtained from chi-square test for categorical variables and t-test for continuous variables

^3^Overlapping categories where some participants experienced more than one listed symptom; chi-square tests were performed for each symptom separately. “Other” symptoms include irritability, numbness in toes, flushed face, cold hands/feet, pins and needles (arms and legs), ringing in ears, tension, and memory loss

^4^Not reported in 22% of participants (*n* = 323/1480); summaries calculated among those with available responses

Table 2 shows measures of pre-injury health disaggregated by sex and age group. More females (58.4%) than males (49.1%; *p* < 0.001) reported previous medical or psychiatric conditions. In particular, 17.8% of females compared to 10.1% of males reported previous psychiatric-related conditions (*p* < 0.001). Participants under 45 years old (i.e., age groups 16–25 yrs and 26–44 yrs) were more likely to be healthy (59.6%) compared to those aged 45 or over (i.e., age groups 45–64 yrs and 65 + years; 29.6%; *p* < 0.001). Recreational drug use was self-reported by more males (5.3%) than females (1.6%; *p* < 0.001) and more younger participants (4.8% of those aged < 45 years *vs*. 2.0% of those ≥ 45 years old; *p* < 0.001). Individuals aged 16–25 years were less than half as likely to use prescription drugs but more than three-fold more likely to use recreational drugs compared to individuals aged ≥ 65 years. The physical component score of the SF-12 decreased with increasing age, whereas the mental component score increased with age. There were no differences in the mental health component, and small, non-clinically significant differences in the physical component, between males and females. Females reported more pre-collision somatic symptoms (mean PHQ-15 = 3.86) than males (mean PHQ-15 = 2.89; *p* < 0.001). No differences were observed between age groups for somatic symptom severity; however, participants under 45 years old had higher pain catastrophizing scores (mean PCS = 8.32) and higher scores for anxiety and depression (mean PHQ-4 = 1.44) than older participants (mean PCS = 6.18, mean PHQ-4 = 0.86; *p* < 0.001).

**Table 2:** Measures of pre-injury health according to sex and age group^1^

	All (*n* = 1,480)	Sex			Age group, yrs				
		Female (*n* = 680)	Male (*n* = 800)	*P*-value^2^	16–25 (*n* = 290)	26–44 (*n* = 549)	45–64 (*n* = 426)	65 + (*n* = 215)	*P*-value^2^
Past medical history^3^									
Healthy	690 (46.6%)	283 (41.6%)	407 (50.9%)	*p* < 0.001	188 (64.8%)	312 (56.8%)	160 (37.6%)	30 (14.0%)	*p* < 0.001
Diabetes	93 (6.3%)	42 (6.2%)	51 (6.4%)	*p* = 0.961	3 (1.0%)	12 (2.2%)	39 (9.2%)	39 (18.1%)	*p* < 0.001
Cardiorespiratory	366 (24.7%)	166 (24.4%)	200 (25.0%)	*p* = 0.830	41 (14.1%)	80 (14.6%)	127 (29.8%)	118 (54.9%)	*p* < 0.001
Psychiatric	202 (13.6%)	121 (17.8%)	81 (10.1%)	*p* < 0.001	46 (15.9%)	82 (14.9%)	58 (13.6%)	16 (7.4%)	*p* = 0.029
Other	478 (32.3%)	255 (37.5%)	223 (27.9%)	*p* < 0.001	40 (13.8%)	124 (22.6%)	168 (39.4%)	146 (67.9%)	*p* < 0.001
Medication use	615 (41.6%)	337 (49.6%)	278 (34.8%)	*p* < 0.001	88 (30.3%)	164 (29.9%)	204 (47.9%)	159 (74.0%)	*p* < 0.001
Recreational drug use	53 (3.6%)	11 (1.6%)	42 (5.3%)	*p* < 0.001	12 (4.1%)	28 (5.1%)	10 (2.3%)	3 (1.4%)	*p* = 0.016
EQ-VAS^4^	86.5 (13.7)	86.5 (14.3)	86.6 (13.2)	*p* = 0.804	87.4 (12.5)	87.1 (13.3)	85.8 (15.4)	85.4 (12.6)	*p* = 0.196
SF-12^5^									
Physical component	52.8 (7.3)	52.3 (7.5)	53.2 (7.1)	*p* = 0.029	54.3 (5.8)	53.5 (6.6)	51.9 (8.0)	50.3 (8.3)	*p* < 0.001
Mental component	53.9 (8.4)	53.7 (8.7)	54.2 (8.1)	*p* = 0.221	51.9 (8.8)	53.2 (8.9)	55.2 (7.6)	56.0 (7.0)	*p* < 0.001
Other health indicators^6^									
PCS total^7^	7.41 (9.35)	7.68 (9.63)	7.18 (9.10)	*p* = 0.317	8.62 (8.70)	8.16 (9.64)	6.84 (9.93)	4.87 (7.57)	*p* < 0.001
Clinically relevant catastrophizing (PCS total > 30)	61 (4.1%)	32 (4.7%)	29 (3.6%)	*p* = 0.298	13 (4.5%)	23 (4.2%)	22 (5.2%)	3 (1.4%)	*p* = 0.113
PHQ-15 total^8^	3.34 (3.71)	3.86 (3.79)	2.89 (3.58)	*p* < 0.001	3.53 (3.59)	3.40 (3.84)	3.22 (3.88)	3.15 (3.14)	*p* = 0.617
High somatic symptom severity (PHQ-15 total > 15)	28 (1.9%)	15 (2.2%)	13 (1.6%)	*p* = 0.448	2 (0.7%)	13 (2.4%)	12 (2.8%)	1 (0.5%)	*p* = 0.063
PHQ-4 total^9^	1.19 (2.25)	1.18 (2.20)	1.20 (2.29)	*p* = 0.886	1.76 (2.56)	1.28 (2.39)	0.94 (2.04)	0.70 (1.57)	*p* < 0.001
Severe psychological distress (PHQ-4 total > 9)	36 (2.4%)	14 (2.1%)	22 (2.8%)	*p* = 0.403	10 (3.4%)	16 (2.9%)	7 (1.6%)	3 (1.4%)	*p* = 0.287

^1^*n* = 1,480 total eligible participants; values are* n* (%) or mean (SD). Where applicable, summaries are based on information collected from participants in the baseline interview

^2^P-values obtained from chi-square test (or Fisher’s exact test) for categorical variables and t-test (or ANOVA test) for continuous variables

^3^Overlapping categories where some participants identify with more than one category; therefore, the sum of reported percentages may exceed 100% and chi-square tests were performed for each category separately (i.e., diabetes vs. no history of diabetes across sex and age groups)

^4^EQ-VAS serves as a measure of health-related quality of life ranking overall health the day before the accident from 0 to 100

^5^The SF-12 questionnaire assesses health-related quality of life 4 weeks prior to the accident. Questionnaire items can be grouped to compute physical and mental component scores which are standardized based on 1998 U.S. population norms, where higher scores suggest better health

^6^Questionnaire scores standardized based on the proportion of total question items answered

^7^The PCS questionnaire assesses the level of pain catastrophizing and coping experienced by the individual, where a higher score suggests a greater level of catastrophizing

^8^The PHQ-15 assesses a variety of somatic symptoms and the degree to which they bother the individual; higher scores suggest a greater level of symptom severity

^9^The PHQ-4 specifically assesses pre-event anxiety and depression, determined based on how often certain problems are experienced by the individual; higher scores suggest greater levels of psychological distress

Table 3 summarizes pre-injury health status according to road user type. Pre-injury medication use was highest among passengers (44.4%) and pedestrians (44.6%) and lowest among cyclists (31.0%) and motorcyclists (39.8%; *p* = 0.035). Passengers self-reported the most severe pre-existing somatic symptoms (mean PHQ-15 = 3.92) and cyclists the least severe (mean PHQ-15 = 2.78; *p* = 0.046). There were no differences in the physical component of the SF-12, and small, non-clinically significant differences for the mental health component, across road user types. No significant differences between road user types were observed for pain catastrophizing or for anxiety and depression. Pre-injury overall health measured using the EQ-VAS was also similar across road user types.

**Table 3:** Measures of pre-injury health status according to road user type^1^

	Driver (*n* = 683)	Passenger (*n* = 225)	Motorcyclist (*n* = 118)	Pedestrian (*n* = 280)	Cyclist (*n* = 174)	*P*-value^2^
Past medical history^3^						
Healthy	318 (46.6%)	108 (48.0%)	57 (48.3%)	113 (40.4%)	94 (54.0%)	*p* = 0.073
Diabetes	45 (6.6%)	14 (6.2%)	6 (5.1%)	24 (8.6%)	4 (2.3%)	*p* = 0.108
Cardiorespiratory	170 (24.9%)	52 (23.1%)	28 (23.7%)	82 (29.3%)	34 (19.5%)	*p* = 0.188
Psychiatric	86 (12.6%)	40 (17.8%)	18 (15.3%)	34 (12.1%)	24 (13.8%)	*p* = 0.319
Other	218 (31.9%)	71 (31.6%)	38 (32.2%)	102 (36.4%)	49 (28.2%)	*p* = 0.457
Medication use	289 (42.3%)	100 (44.4%)	47 (39.8%)	125 (44.6%)	54 (31.0%)	*p* = 0.035
EQ-VAS^4^	86.94 (13.68)	85.15 (15.48)	87.34 (9.89)	86.14 (14.99)	86.94 (10.96)	*p* = 0.457
SF-12^5^						
Physical component	52.8 (7.1)	51.7 (8.1)	53.8 (6.1)	52.7 (7.4)	53.6 (7.2)	*p* = 0.054
Mental component	54.4 (8.2)	52.5 (9.6)	52.9 (8.4)	54.5 (7.9)	53.9 (8.0)	*p* = 0.025
Other health indicators^6^						
PCS total^7^	7.42 (9.48)	8.38 (10.31)	7.15 (8.61)	7.31 (9.40)	6.48 (7.81)	*p* = 0.391
Clinically relevant catastrophizing (PCS > 30)	29 (4.2%)	14 (6.2%)	3 (2.5%)	12 (4.3%)	3 (1.7%)	*p* = 0.200
PHQ-15 total^8^	3.36 (3.66)	3.92 (4.12)	3.14 (3.38)	3.24 (3.89)	2.78 (3.11)	*p* = 0.046
High somatic symptom severity (PHQ-15 > 15)	13 (1.9%)	5 (2.2%)	1 (0.8%)	8 (2.9%)	1 (0.6%)	*p* = 0.499
PHQ-4 total^9^	1.28 (2.36)	1.39 (2.40)	0.91 (1.69)	0.99 (2.06)	1.12 (2.22)	*p* = 0.133
Severe psychological distress (PHQ-4 > 9)	19 (2.8%)	7 (3.1%)	0 (0.0%)	6 (2.1%)	4 (2.3%)	*p* = 0.386

^1^*n* = 1,480 total eligible participants; values are* n* (column %) or mean (SD)

^2^P-value obtained from chi-square test (or Fisher’s exact test) for categorical variables and ANOVA test for continuous variables

^3^Overlapping categories where some participants identify with more than one category; therefore, the sum of reported percentages exceed 100% and chi-square tests were performed for each category separately (i.e., diabetes vs. no diabetes across road user type)

^4^EQ-VAS serves as a measure of health-related quality of life ranking overall health the day before the accident from 0 to 100

^5^The SF-12 questionnaire assesses health-related quality of life 4 weeks prior to the accident. Questionnaire items can be grouped to compute physical and mental component scores which are standardized based on 1998 U.S. population norms, where higher scores suggest better health

^6^Questionnaire scores standardized based on the proportion of total question items answered

^7^The PCS questionnaire assesses the level of pain catastrophizing and coping experienced by the individual, where a higher score suggests a greater level of catastrophizing

^8^The PHQ-15 assesses a variety of somatic symptoms and the degree to which they bother the individual; higher scores suggest a greater level of symptom severity

^9^The PHQ-4 specifically assesses pre-event anxiety and depression, determined based on how often certain problems are experienced by the individual; higher scores suggest greater levels of psychological distress

Table 4 summarizes demographics, injury details and related symptoms, and recovery expectations according to road user type. Drivers and pedestrians were older (mean age = 43.5 and 46.9 years, respectively) than other road users (mean age = 40.6, 40.8, 40.1 years for passengers, motorcyclists, and cyclists, respectively; *p* < 0.001). Approximately half of the drivers (48.6%) and pedestrians (49.6%) were female, whereas most motorcyclists (83.9%) and cyclists (76.4%) were male. Distribution of ISS by road user type is displayed in Fig. 2. Motorcyclists (mean ISS = 10.3) and pedestrians (mean ISS = 10.7) sustained more severe injury than drivers (mean ISS = 5.72), passengers (mean ISS = 6.08) or cyclists (mean ISS = 7.11; *p* < 0.001). Despite injury severity scores, self-reported pain severity was highest among pedestrians and motor vehicle passengers (mean pain severity = 6.90 and 6.69, respectively) and lowest among cyclists and motorcyclists (mean pain severity = 5.99 and 5.82, respectively; *p* < 0.001). There were statistically significant differences in injury location between road user groups. Head injuries were most prevalent in pedestrians (47.5%) and passengers (42.7%) and least prevalent in motorcyclists (19.5%). Upper extremity injuries were most prevalent in cyclists (66.1%) and motorcyclists (62.7%) and least prevalent in passengers (39.6%) and drivers (45.8%). Lower extremity injuries were most prevalent in motorcyclists (75.4%) and pedestrians (72.1%) and least prevalent in drivers (32.2%) and passengers (33.8%). In terms of injury symptoms, motor vehicle occupants (drivers and passengers) were more likely to report headache, dizziness, chest pain, back and neck pain and stiffness following accident compared to other road user types. Interestingly, motorcyclists and cyclists were more likely to have an idea of their recovery trajectory, 45–50% of motorists or pedestrians did not know how long they would take to fully recover from injuries compared to 24% for motorcyclists and 31% for cyclists.

**Table 4:** Injury details and recovery expectations according to road user type^1^

	Driver (*n* = 683)	Passenger (*n* = 225)	Motorcyclist (*n* = 118)	Pedestrian (*n* = 280)	Cyclist (*n* = 174)	*P*-value^2^
Age, yrs	43.51 (18.17)	40.64 (19.39)	40.78 (14.85)	46.93 (19.90)	40.10 (15.15)	*p* < 0.001
Female	332 (48.6%)	149 (66.2%)	19 (16.1%)	139 (49.6%)	41 (23.6%)	*p* < 0.001
Disposition						
Admitted	116 (17.0%)	42 (18.7%)	50 (42.4%)	110 (39.3%)	38 (21.8%)	*p* < 0.001
Discharged	557 (81.6%)	180 (80.0%)	67 (56.8%)	167 (59.6%)	134 (77.0%)	
Injury severity score	5.72 (8.69)	6.08 (9.49)	10.03 (9.95)	10.07 (10.30)	7.11 (8.37)	*p* < 0.001
Pain severity (0–10)^3^	6.39 (2.49)	6.69 (2.43)	5.82 (2.45)	6.90 (2.63)	5.99 (2.11)	*p* < 0.001
Injury location^4^						
Head (skull, brain)	254 (37.2%)	96 (42.7%)	23 (19.5%)	133 (47.5%)	61 (35.1%)	*p* < 0.001
Neck	362 (53.0%)	99 (44.0%)	21 (17.8%)	48 (17.1%)	28 (16.1%)	*p* < 0.001
Chest	201 (29.4%)	66 (29.3%)	34 (28.8%)	59 (21.1%)	25 (14.4%)	*p* < 0.001
Abdomen/pelvis	92 (13.5%)	45 (20.0%)	29 (24.6%)	70 (25.0%)	44 (25.3%)	*p* < 0.001
Spine/back	277 (40.6%)	82 (36.4%)	27 (22.9%)	68 (24.3%)	47 (27.0%)	*p* < 0.001
Upper extremity	313 (45.8%)	89 (39.6%)	74 (62.7%)	153 (54.6%)	115 (66.1%)	*p* < 0.001
Lower extremity	220 (32.2%)	76 (33.8%)	89 (75.4%)	202 (72.1%)	118 (67.8%)	*p* < 0.001
Symptoms immediately after accident^4^						
Headache	235 (34.4%)	76 (33.8%)	17 (14.4%)	67 (23.9%)	30 (17.2%)	*p* < 0.001
Chest pain	168 (24.6%)	64 (28.4%)	25 (21.2%)	35 (12.5%)	21 (12.1%)	*p* < 0.001
Back pain/stiff back	293 (42.9%)	90 (40.0%)	40 (33.9%)	85 (30.4%)	58 (33.3%)	*p* = 0.002
Neck pain/stiff neck	344 (50.4%)	91 (40.4%)	28 (23.7%)	78 (27.9%)	44 (25.3%)	*p* < 0.001
Shortness of breath	188 (27.5%)	70 (31.1%)	25 (21.2%)	52 (18.6%)	48 (27.6%)	*p* = 0.009
Dizziness	257 (37.6%)	77 (34.2%)	24 (20.3%)	81 (28.9%)	50 (28.7%)	*p* < 0.001
Other	546 (79.9%)	186 (82.7%)	95 (80.5%)	236 (84.3%)	155 (89.1%)	*p* = 0.058
Recovery expectation^3^						
< 1 week	74 (13.4%)	25 (14.0%)	9 (9.4%)	16 (8.1%)	13 (9.8%)	*p* < 0.001
1 week to < 1 month	118 (21.3%)	38 (21.2%)	22 (22.9%)	39 (19.8%)	41 (31.1%)	
1 month to < 3 months	55 (9.9%)	14 (7.8%)	19 (19.8%)	21 (10.7%)	17 (12.9%)	
3 months to < 6 months	23 (4.2%)	6 (3.4%)	10 (10.4%)	11 (5.6%)	9 (6.8%)	
≥ 6 months	29 (5.2%)	14 (7.8%)	13 (13.5%)	11 (5.6%)	11 (8.3%)	
Don’t know	253 (45.8%)	82 (45.8%)	23 (24.0%)	99 (50.3%)	41 (31.1%)	

^1^*n* = 1,480 total eligible participants; values are* n* (column %) or mean (SD)

^2^P-value obtained from chi-square test for categorical variables and ANOVA test for continuous variables

^3^Not reported in 22% of participants (*n* = 323/1480); summaries calculated among those with available responses

^4^Overlapping categories; therefore, the sum of reported percentages exceeds 100% and chi-square tests were performed for each injury location and symptom separately. “Other” symptoms include irritability, numbness in toes, flushed face, cold hands/feet, pins and needles (arms and legs), ringing in ears, tension, and memory loss

## Discussion

We prospectively enrolled 1,480 road trauma survivors who were treated for injuries in three Canadian trauma centres. Approximately half of the drivers and pedestrians and two thirds of the passengers were female, whereas the majority of motorcyclists and cyclists were male. These results are consistent with an Australian study of road trauma survivors, which found injured motorcyclists and bicyclists were predominantly male (88.1% and 75.9%, respectively) while 46.7% of vehicle occupants were male [49]. The large proportion of male motorcyclists and cyclists is likely because these transportation modes are used more often by males than females [50–53]. Male cyclists may be also more likely to be involved in a crash due to a higher prevalence of cycling at night, under the influence of alcohol, or at high speed [54, 55]. Consistent with this hypothesis, a higher percentage of males than females in our cohort reported recreational drug use. The nearly equal numbers of male and female motor vehicle drivers differs from most hospital studies of injured drivers which are typically about two thirds male [56]. This may be because our cohort included minor injury crashes; the percentage of female drivers is higher in minor crashes than in serious crashes [57]. We found that pedestrians were older than other road users, probably because older pedestrians are at higher risk of collision due to reduced cognitive and visual abilities, resulting in decision-making difficulties (e.g., during street crossing), especially when under time pressure [58]. In addition, elderly pedestrians are at risk of sustaining more severe injuries and worse outcomes after a collision has occurred because of medical frailty [59].

An injury severity score (ISS) ≥ 15 is associated with 10% mortality and is commonly used to define ‘major’ or ‘severe’ trauma [60]. Our cohort had relatively low injury severity, the mean (SD) ISS in this sample was 7.11 (9.37) and only a quarter required admission to hospital. Injury severity and need for hospital admission was higher in males, possibly due to increased risk-taking and higher probability of being involved in high-speed collisions [61]. Injury severity also varied according to road user type, with higher injury severity and need for hospital admission among pedestrians and motorcyclists. Previous studies also noted higher injury severity in motorcyclists [49]. Although pedestrians, motorcyclists and cyclists are all vulnerable road users who are at greater risk of severe injury in event of a collision, we found that cyclists had lower injury severity and less need for hospital admission compared to pedestrians and motorcyclists. This may be because many cyclist-motorist collisions occur at low speed (e.g., car makes a turn and cuts the cyclist off). Additionally, as cyclists were younger and healthier than pedestrians they may be less likely to be severely injured in a collision. Motorcyclists, on the other hand, travel at higher speeds, increasing the risk for more serious injury. These findings may also be explained by recent improvements in cycling safety in British Columbia such as improved cycling infrastructure with separated bicycle lanes and promotion of behavioural changes in motorists to increase their awareness of cyclists sharing the road. Given the growing uptake of active transportation, the severity of road trauma among pedestrians highlights the need for changes to the built environment to improve pedestrian safety. Pedestrian injuries can be decreased with traffic control changes (such as leading pedestrian intervals), raised medians on multilane roads, and measures to reduce vehicle turning speeds [62–66].

Significant differences in injury location were observed between road user types. Extremity injuries were most common in vulnerable road users (pedestrians, motorcyclists and cyclists). Head injuries were most common in pedestrians and least common in motorcyclists and cyclists. This could have been a result of helmet use by motorcyclists and cyclists protecting them from more severe head injury. In addition, since cyclists and motorcyclists were younger, they were likely more able to react and protect their heads as they fell following the collision.

Overall, this cohort of road trauma survivors reported good pre-injury health. Males reported higher SF-12 physical component scores but there was no significant difference between males and females with respect to mental component scores. As expected, the physical component score of the SF-12 decreased with increasing age suggesting poorer physical health in older participants; however, the mental component score increased with age. This paradoxical trend of deterioration of physical and cognitive functioning but improvement in various attributes of mental health with age has been noted in other studies and attributed to greater resilience to common physical and social stresses, increased wisdom, and greater life satisfaction with increasing age [67]. In terms of road user type, motorcyclists and cyclists reported better physical health through the SF-12 whereas motor vehicle drivers and pedestrians had higher mental component scores. Approximately half of the 1,480 road trauma survivors reported having no significant past medical conditions, with a greater representation of “healthy” individuals among cyclists, males, and the younger age groups (16–25 and 26–44 years). Cyclists and pedestrians reported better overall health than motorists, but the differences were not as substantial as expected considering the known health benefits of active transport [27].

Since recovery from injury is influenced by psychological factors, we used validated measures to assess pre-injury pain catastrophizing (PCS), somatic symptomatology (PHQ-15), and psychological distress (PHQ-4). Pain catastrophizing, defined as persistent negative cognitive and emotional responses to actual or anticipated pain, [46] undermines behavioural and medical treatments [68, 69]. High catastrophizing levels (PCS ≥ 30) [46] are hypothesized to be associated with poor recovery following injury. According to the *good old days bias*, injury survivors often underestimate their pre-injury symptoms and may retrospectively attribute pre-existing symptoms to the injury itself [70]. Therefore, people with high pre-injury PHQ-15 scores (≥ 15) [47] may report poor recovery if they attribute pre-existing somatic symptoms to the injury. Our cohort had minimal levels of pain catastrophizing, mild levels of somatic symptoms, and minimal levels of psychological distress prior to the accident, with less than 5% of the cohort experiencing severe symptoms. Females reported higher severity of somatic symptoms and greater pain severity and more commonly reported headache or shortness of breath. This is consistent with previous literature suggesting women report more intense and more frequent bodily symptoms, possibly due to innate differences in somatic and visceral perception or differences in symptom labeling and reporting [71]. Consistent with trends in the SF-12 mental component score, the level of pain catastrophizing (PCS) and severity of anxiety and depression (PHQ-4) also decreased with increasing age. Compared to other road users, motorcyclists and cyclists reported lower severity of somatic symptoms and also self-reported lower pain severity, whereas motor vehicle occupants were more likely to report headache, dizziness, chest pain, back/neck pain and stiffness symptoms.

This study was designed to overcome many limitations of previous road trauma research and has many strengths. A large inception cohort of almost 1,500 road trauma survivors was recruited. Inception cohorts are less prone to sampling and recall bias compared to retrospective cohorts [72, 73]. In addition, to maximize generalizability, restrictions on recruitment were not placed on road user type, injury severity level, or language; however, 89 of 2,618 potentially eligible participants were excluded because of language barrier and no available translator. Patient-reported outcomes were collected through validated tools to study HRQoL from both physical and psychological domains. In addition, since previous road trauma outcome research in North America is limited, this study addresses the limited generalizability of previous research; cultural variation exists for many risk factors for poor recovery, including recovery expectations and crash severity perception [36].

Our study has several limitations. As we relied on self-reported tools, we acknowledge that recall and reporting bias may be present, especially regarding report of pre-injury health status. The *good old days bias*, a type of recall bias where individuals misremember and tend to exaggerate their pre-injury HRQoL, is common following injury [74–76]. Efforts were made to try to minimize this bias by conducting baseline interviews as soon as possible following the crash, within 7 days in most cases (> 90%). Reasons for delay of interview included severe injury, such as a head injury or one that resulted in an ICU stay, where individuals were unable to communicate or were unable to be reached. We acknowledge the limitation that recollection of injury details and pre-event health status would be decreased in such cases of more severe injury. Additionally, some outcomes reported such as assessment of pain severity on a scale from 0–10 may not be conducive to proxy reporting; however, proxy reporting was rare, occurring in only 0.8% of participant baseline questionnaires (*n* = 12/1480). Non-response bias may have also been present which may limit our ability to compare road user groups. Older individuals were less likely to participate than younger individuals and cyclists were more likely to participate than other road users. Individuals with very minor injuries may have been discharged more rapidly from the ED before they could be approached by research staff. Conversely, some individuals declined participation because they had too much pain or discomfort to be interviewed. Although interviews were offered in multiple languages, we may have missed some non-English speaking individuals. Additionally, road trauma survivors who never sought hospital care were missed.

### Future directions

The next step in this research will be to study the one-year outcome of cohort participants and identify pre- and post-injury factors associated with different recovery trajectories. The simple self-reported scales used to assess pre-injury health in this cohort could be used clinically to assess self-reported pre-injury health in injured patients, including patients with minor injuries who are not usually included in trauma registries; even minor injury can result in prolonged pain and/or disability in some patients. Routine collection of baseline health data on all injured patients, combined with self-reported recovery data, will provide insight into why some patients have better recovery than others. This information could help clinicians identify patients at risk of poor recovery. Additionally, the severity of trauma identified among vulnerable road users suggests an opportunity for improvements in city planning and the built environment surrounding these individuals, including infrastructure for separation from other more protected road user types.

## Conclusions

We present a comprehensive overview of sociodemographic and injury characteristics of a large cohort of road trauma survivors presenting to the ED. Inception cohorts of this size are relatively uncommon and information on baseline characteristics is not well documented especially in a North American setting. Overall injury severity for this cohort was low. Motorcyclists and pedestrians, but not cyclists, had more severe injuries than motorists. Extremity injuries were more common in vulnerable road users. Recovery following road trauma depends on both injury type and severity but also on pre-injury health and psychological factors. Future research will investigate one-year recovery outcomes of this cohort and identify factors associated with poor recovery following road traffic injury.

### Supplementary Information

**Additional file 1: Table S1. **Comparison of characteristics of eligible individuals who refused participation and those who were enrolled. **Table S2. **Sociodemographic characteristics of the enrolled cohort. 

## Data Availability

The datasets generated and/or analyzed during the current study are not publicly available due to privacy issues but are available from the corresponding author on reasonable request.

## Abbreviations

PTSD: Post-traumatic stress disorder
HRQoL: Health-related quality of life
ED: Emergency department
PHQ-4: Patient Health Questionnaire-4
PHQ-15: Patient Health Questionnaire-15
PCS: Pain Catastrophizing Scale
SF-12: 12-Item Short Form Health Survey
VAS: Visual analogue scale
VGH: Vancouver General Hospital
RCH: Royal Columbian Hospital
KGH: Kelowna General Hospital
ISS: Injury severity score
