# Supplementary material for: Demographic and clinical profile of an inception cohort of road trauma survivors
Source: BMC Public Health. 2023 Aug 12;23:1534. doi: 10.1186/s12889-023-16487-w (PMC10422727; doi:10.1186/s12889-023-16487-w)
Supplement: Supplementary file 1 — Additional file 1: Table S1. Comparison of characteristics of eligible individuals who refused participation and those who were enrolled. Table S2. Sociodemographic characteristics of the enrolled cohort. [file 12889_2023_16487_MOESM1_ESM.docx]

**SUPPLEMENTARY TABLES**

**Table S1** Comparison of characteristics of eligible individuals who refused participation and those who were enrolled^1^

|  | Eligible but refused  (*n*=1,138) | Eligible and enrolled  (*n*=1,480) | P-value^2^ |
| --- | --- | --- | --- |
| Site |  |  |  |
| VGH | 727 (63.9%) | 1,219 (82.4%) | p<0.001 |
| RCH | 273 (24.0%) | 219 (14.8%) |  |
| KGH | 138 (12.1%) | 42 (2.8%) |  |
| Age, yrs | 46.1 (19.1) | 43.1 (18.3) | p<0.001 |
| Sex^3^ |  |  |  |
| Female | 331 (45.8%) | 680 (45.9%) | p=0.995 |
| Male | 392 (54.2%) | 800 (54.1%) |  |
| Road user type |  |  |  |
| Driver | 568 (51.3%) | 683 (46.1%) | p<0.001 |
| Passenger | 176 (15.9%) | 225 (15.2%) |  |
| Motorcyclist | 90 (8.1%) | 118 (8.0%) |  |
| Cyclist | 58 (5.2%) | 174 (11.8%) |  |
| Pedestrian | 215 (17.6%) | 280 (18.9%) |  |
| Disposition |  |  |  |
| Discharged home | 819 (72.0%) | 1,105 (74.7%) | p=0.228 |
| Admitted to hospital | 304 (26.7%) | 356 (24.1%) |  |
| Left against medical advice / without being seen / before treatment completed | 15 (1.3%) | 15 (1.0%) |  |

^1^ Values are *n* (column %) or mean (SD). VGH, Vancouver General Hospital; RCH, Royal Columbian Hospital; KGH, Kelowna General Hospital.

^2^ P-values obtained from chi-square test for categorical variables and t-test for continuous variables.

^3^ Sex distribution in “eligible but refused” group based on subset of participants screened at VGH (total *n*=723).

**Table S2** Sociodemographic characteristics of the enrolled cohort^1^

|  | All  (*n*=1,480) | Sex | | |
| --- | --- | --- | --- | --- |
|  |  | Female  (*n*=680) | Male  (*n*=800) | P-value^2^ |
| Age, yrs |  |  |  |  |
| 16–25 | 290 (19.6%) | 135 (19.9%) | 155 (19.4%) | p=0.461 |
| 26–35 | 356 (24.1%) | 153 (22.5%) | 203 (25.4%) |  |
| 36–45 | 221 (14.9%) | 99 (14.6%) | 122 (15.3%) |  |
| 46–55 | 211 (14.3%) | 93 (13.7%) | 118 (14.8%) |  |
| 56–65 | 197 (13.3%) | 92 (13.5%) | 105 (13.1%) |  |
| 66–74 | 109 (7.4%) | 56 (8.2%) | 53 (6.6%) |  |
| 75+ | 96 (6.5%) | 52 (7.6%) | 44 (5.5%) |  |
| Education level |  |  |  |  |
| Less than high school | 96 (6.5%) | 34 (5.0%) | 62 (7.8%) | p=0.003 |
| High school | 419 (28.3%) | 175 (25.7%) | 244 (30.5%) |  |
| Vocational education | 150 (10.1%) | 60 (8.8%) | 90 (11.3%) |  |
| Post-secondary  (bachelor’s) | 611 (41.3%) | 303 (44.6%) | 308 (38.5%) |  |
| Post-secondary (master’s/doctoral) | 187 (12.6%) | 98 (14.4%) | 89 (11.1%) |  |
| Employment status |  |  |  |  |
| Employed | 981 (66.3%) | 399 (58.7%) | 582 (72.8%) | p<0.001 |
| Homemaker | 17 (1.1%) | 16 (2.4%) | 1 (0.1%) |  |
| Unemployed | 80 (5.4%) | 35 (5.1%) | 45 (5.6%) |  |
| Going to school | 154 (10.4%) | 87 (12.8%) | 67 (8.4%) |  |
| Retired | 200 (13.5%) | 119 (17.5%) | 81 (10.1%) |  |
| Ethnicity |  |  |  |  |
| Caucasian | 739 (49.9%) | 308 (45.3%) | 431 (53.9%) | p<0.001 |
| East Asian | 229 (15.5%) | 134 (19.7%) | 95 (11.9%) |  |
| South Asian | 135 (9.1%) | 59 (8.7%) | 76 (9.5%) |  |
| African American | 24 (1.6%) | 8 (1.2%) | 16 (2.0%) |  |
| Indigenous | 41 (2.8%) | 22 (3.2%) | 19 (2.4%) |  |
| Other | 230 (15.5%) | 111 (16.3%) | 119 (14.9%) |  |
| Multiple^3^ | 62 (4.2%) | 28 (4.1%) | 34 (4.3%) |  |
| Years lived in Canada |  |  |  |  |
| Entire life | 789 (53.3%) | 332 (48.8%) | 457 (57.1%) | p=0.021 |
| More than 10 years | 463 (31.3%) | 235 (34.6%) | 228 (28.5%) |  |
| 5 to 10 years | 89 (6.0%) | 39 (5.7%) | 50 (6.3%) |  |
| 2 to 5 years | 68 (4.6%) | 37 (5.4%) | 31 (3.9%) |  |
| <2 years | 52 (3.5%) | 26 (3.8%) | 26 (3.3%) |  |
| Living situation |  |  |  |  |
| Live alone | 351 (23.7%) | 157 (23.1%) | 194 (24.3%) | p=0.098 |
| Live with partner and/or children | 713 (48.2%) | 342 (50.3%) | 371 (46.4%) |  |
| Live with family or friends | 341 (23.0%) | 141 (20.7%) | 200 (25.0%) |  |
| Other | 52 (3.5%) | 29 (4.3%) | 23 (2.9%) |  |

^1^ Values are *n* (column % by variable category).

^2^ P-values obtained from chi-square test for categorical variables.

^3^ Denotes category for participants who self-identify with more than one ethnicity.
